# Acute Exacerbation of Chronic Cholecystitis Revealing a Duplicate Gallbladder: A Rare Surgical Challenge

**DOI:** 10.7759/cureus.91140

**Published:** 2025-08-27

**Authors:** Mark Salib, John Salib, Almir Music, Elias Kondilis, Matthew Phillips

**Affiliations:** 1 School of Medicine, St. George's University School of Medicine, St. George's, GRD; 2 General Surgery, Community First Medical Center, Chicago, USA

**Keywords:** acute calculus cholecystitis, duplicate gallbladder, laparoscopic surgery, lap chole, rare congenital anomaly

## Abstract

Gallbladder duplication is a rare congenital anomaly that may complicate the diagnosis and surgical management of biliary disease. We report the case of a 42-year-old gentleman presenting with the sudden onset of right upper abdominal pain, nausea, and emesis. Imaging revealed a duplicated gallbladder with cholelithiasis and sonographic features consistent with acute cholecystitis. The patient underwent a laparoscopic cholecystectomy, which revealed a bilobed gallbladder with a single cystic duct. This case highlights the diagnostic and operative challenges associated with gallbladder duplication and emphasizes the importance of preoperative recognition to ensure safe and effective surgical intervention.

## Introduction

Gallbladder duplication is a rare congenital anomaly of the biliary system, occurring in roughly one in 4,000 individuals [1]. It results from an abnormal budding of the hepatic diverticulum during early embryonic development, typically between the fifth and sixth week of gestation [2,3]. This anomaly results in the development of two gallbladder structures, which may vary significantly in their anatomical separation and vascular supply. The widely used Harlaftis classification divides these anomalies into two main types: type I, where a single cystic duct drains a bilobed or septated gallbladder, and type II, which involves two completely separate gallbladders, each with its own cystic duct [4].

The most duplicated gallbladders are asymptomatic and discovered incidentally. However, when one becomes inflamed or develops stones, the condition quickly becomes clinically significant [5]. Diagnosis is often challenging, as the duplicated anatomy may be misinterpreted as other biliary anomalies, such as a folded gallbladder, choledochal cyst, or pericholecystic fluid collection [6]. Due to the rarity of this condition, it is frequently recognized intraoperatively, increasing the risk for complications such as bile duct injury or incomplete resection if both components are not adequately identified [7].

Thus, in this report, we describe a rare case of type I gallbladder duplication complicated by acute cholecystitis and cholelithiasis. By outlining the clinical presentation, imaging findings, surgical approach, and pathology, we aim to underscore the importance of early recognition of this unusual anatomy. A thorough understanding of these anomalies is essential for safe and effective surgical planning.

## Case presentation

A 42-year-old gentleman presented to the emergency department with right upper and lower abdominal pain, accompanied by nausea and multiple episodes of emesis. The symptoms began the previous evening and had progressively worsened. He reported experiencing three similar episodes in the past year, but did not seek medical attention as they were not as severe. He denied fever, chills, shortness of breath, chest pain, palpitations, recent illness, changes in bowel habits, or unintentional weight loss. His medical history was notable for a laparoscopic appendectomy performed in 2021.

On physical examination, the patient appeared mildly fatigued. Vital signs were within normal limits. Abdominal examination revealed a soft, non-distended abdomen with localized tenderness to both superficial and deep palpation in the right upper quadrant. Hypoactive bowel sounds (less than five bowel sounds per minute) and a positive Murphy’s sign were noted.

Laboratory studies were notable for leukocytosis of 14,900/µL, suggesting an underlying inflammatory or infectious process. The patient’s hemoglobin and platelet levels were within normal limits. Liver function tests revealed alanine aminotransferase (ALT), aspartate aminotransferase (AST), total bilirubin, and alkaline phosphatase within reference ranges. Renal function, as assessed by blood urea nitrogen (BUN) and creatinine levels, was within normal limits. Serum electrolytes, amylase, and lipase were unremarkable. These findings collectively supported the clinical suspicion of acute gallbladder pathology without evidence of hepatic or pancreatic involvement. A complete summary of laboratory values is provided in Table 1.

**Table 1:** Summary of the initial laboratory evaluation on admission Comprehensive laboratory panel demonstrating leukocytosis (WBC 14.9 × 10³/µL) with otherwise unremarkable complete blood count. Basic metabolic panel reveals mildly elevated fasting glucose (118 mg/dL) with normal renal function and electrolytes. Liver function tests are within normal limits. These findings are consistent with a mild inflammatory response and preserved hepatic and renal function

Category	Test (abbreviation)	Value	Reference Range
Inflammatory/pancreatic	C-reactive protein (CRP)	5	<10 mg/L
	Erythrocyte sedimentation rate (ESR)	10	0-20 mm/hr (male), 0-30 mm/hr (female)
	Amylase	70	30-110 U/L
	Lipase	50	10-140 U/L
Complete blood count (CBC)	White blood cells (WBC)	14.9	4.0-11.0 × 10³/µL
	Red blood cells (RBC)	5.48	4.2-5.9 × 10⁶/µL
	Hemoglobin (HGB)	15.8	13.5-17.5 g/dL
	Hematocrit (HCT)	45.7	38.8-50.0%
	Platelets (PLT)	304	150-450 × 10³/µL
Basic metabolic panel (BMP)	Glucose (GLU)	118	70-99 mg/dL (fasting)
	Sodium (Na)	140	135-145 mmol/L
	Potassium (K)	3.8	3.5-5.1 mmol/L
	Chloride (Cl)	106	98-107 mmol/L
	Carbon dioxide (CO₂)	28	23-30 mmol/L
	Blood urea nitrogen (BUN)	18	7-20 mg/dL
	Creatinine (CREATININE)	1.06	0.6-1.3 mg/dL
	BUN-to-creatinine ratio (BCR)	17	10:1-20:1
	Calcium (Ca)	8.8	8.6-10.3 mg/dL
Liver function tests (LFTs)	Total protein (PROT)	6	6.0-8.3 g/dL
	Albumin (ALBUMIN)	3.7	3.5-5.0 g/dL
	Aspartate aminotransferase (AST)	13	10-40 U/L
	Alanine aminotransferase (ALT)	13	7-56 U/L
	Alkaline phosphatase (ALKPHOS)	35	40-129 U/L
	Total bilirubin (BILITOT)	0.7	0.1-1.2 mg/dL
White blood cell differential (WBC diff)	Neutrophils	11.2	1.8-7.7 × 10³/µL
	Lymphocytes	2.2	1.0-4.8 × 10³/µL
	Monocytes	1	0.2-1.0 × 10³/µL
	Eosinophils	0.3	0-0.5 × 10³/µL
	Basophils	0.15	0-0.2 × 10³/µL

**Figure 1:**
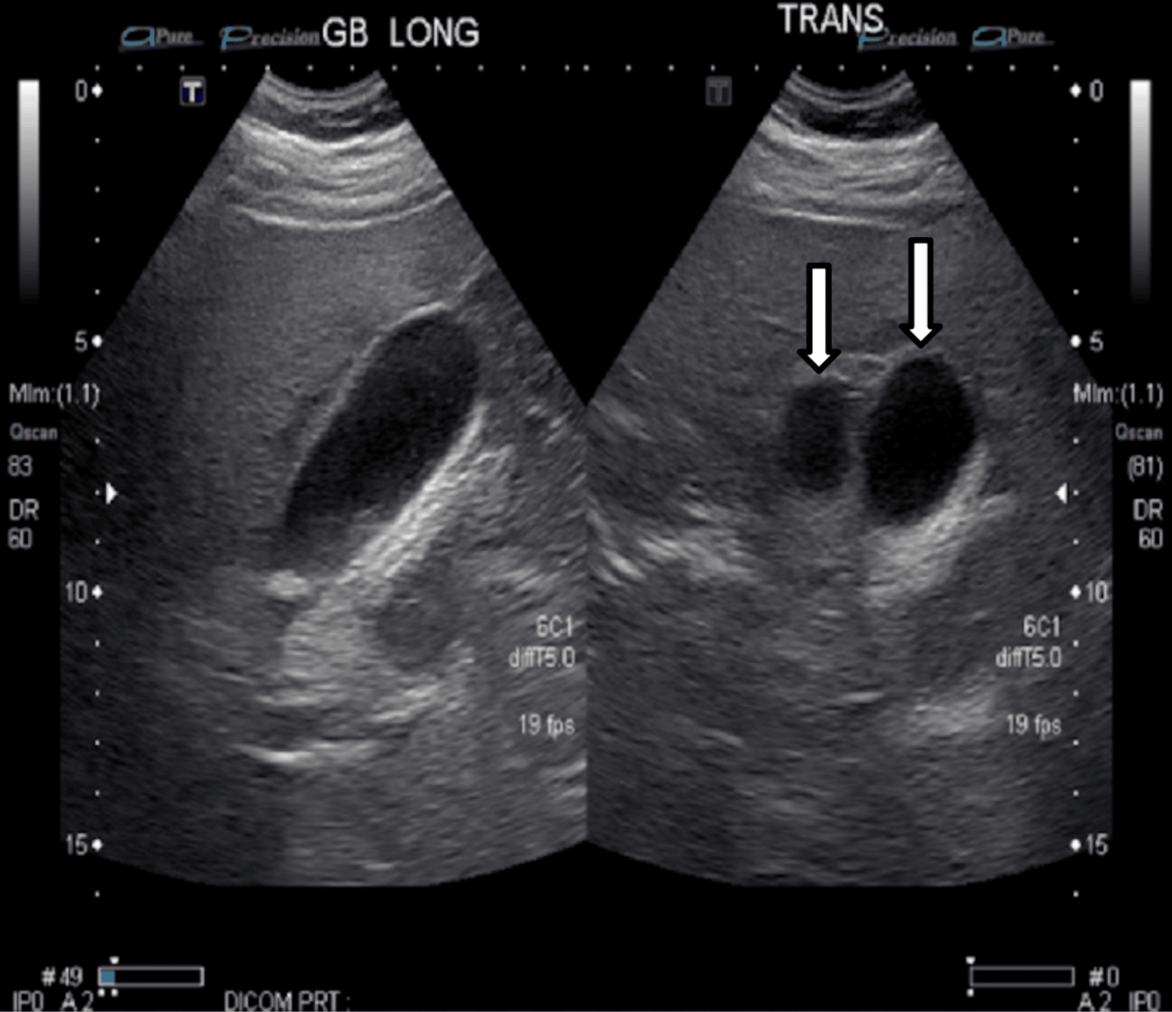
Ultrasound images demonstrating duplicated gallbladder with features of acute cholecystitis Longitudinal (GB LONG) and transverse (TRANS) grayscale ultrasound images of the right upper quadrant demonstrate two distinct anechoic structures consistent with a duplicated gallbladder (as depicted by the white arrows). The walls appear thickened, and internal echoes suggest sludge or debris, supporting a diagnosis of acute cholecystitis

**Figure 2:**
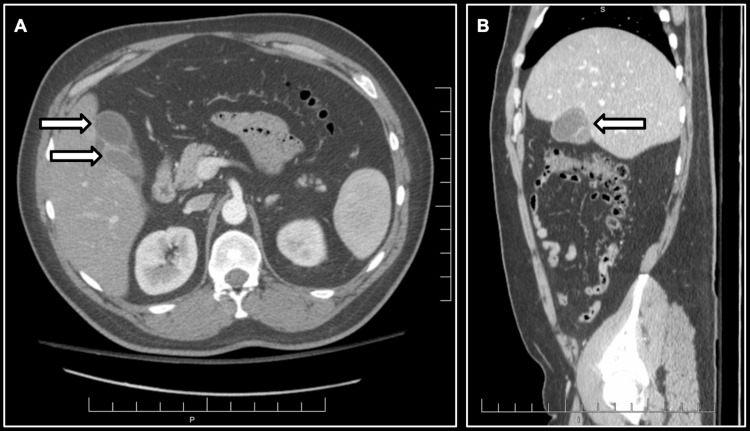
CT imaging demonstrating gallbladder duplication with acute cholecystitis (A) Axial contrast-enhanced CT image of the abdomen shows two fluid-filled, tubular structures in the right upper quadrant (white arrows), located in the gallbladder fossa. (B) Coronal reformatted CT image shows the duplicated gallbladders oriented vertically beneath the liver (white arrow), confirming their distinct morphology and alignment

An abdominal ultrasound (Figure 1) revealed two distinct gallbladder-like structures consistent with gallbladder duplication, in addition to cholelithiasis and biliary sludge. These findings were confirmed on contrast-enhanced computed tomography (CT) of the abdomen and pelvis (Figure 2), which also demonstrated radiographic features consistent with acute cholecystitis involving one of the duplicated gallbladders.

**Figure 3:**
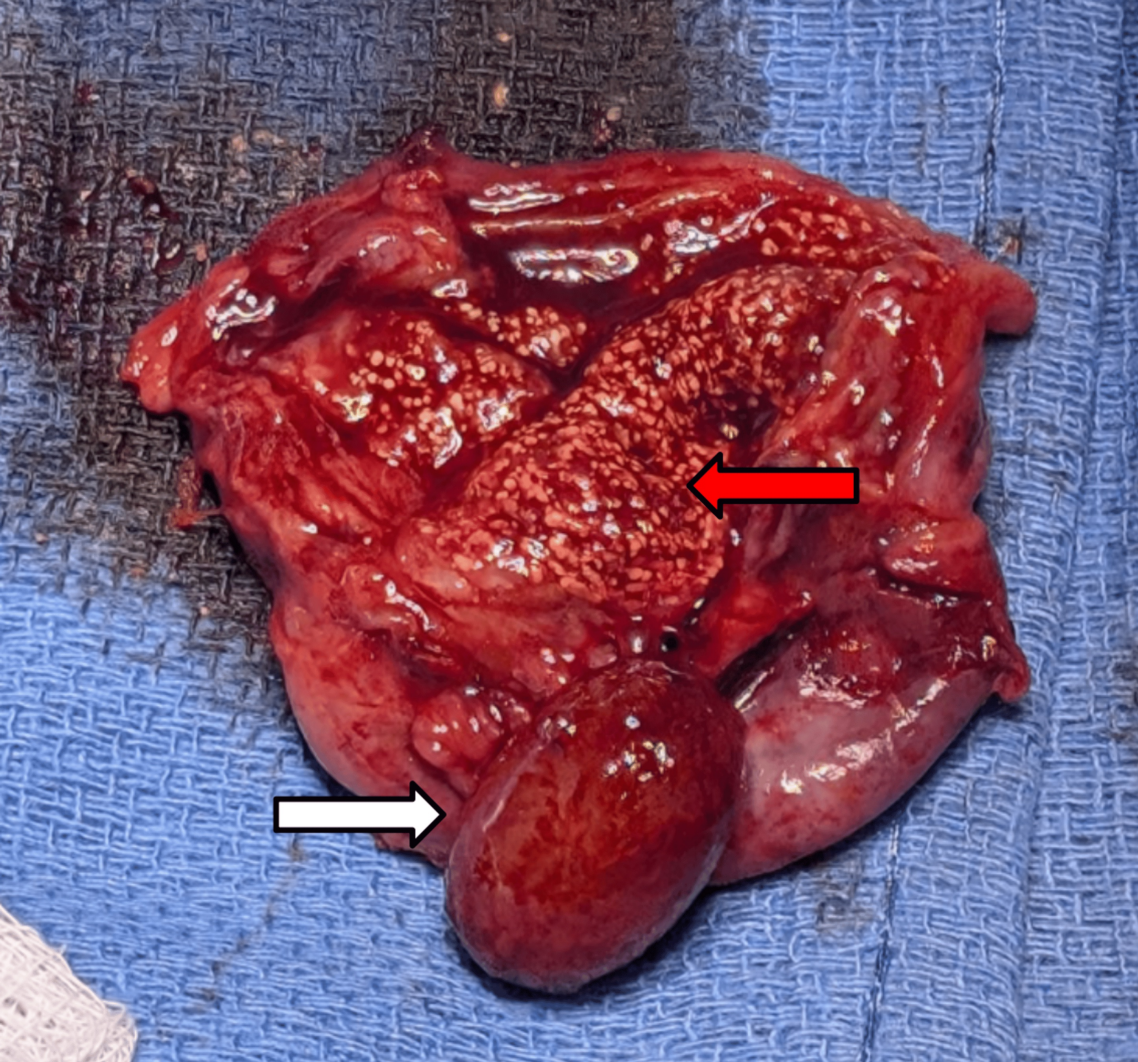
Gross specimen of the resected gallbladder The specimen shows an open gallbladder measuring 6.6 cm in length and 4.8 cm in circumference. The lumen contains minimal yellow bile along with a single yellow calculus (as depicted by the white arrow) measuring 2.0 × 1.2 × 1.2 cm, which reveals a yellow-brown, crystalline interior upon inspection. The gallbladder wall measures up to 0.4 cm in thickness, and the mucosal surface appears mildly roughened (as depicted by the red arrow)

A diagnosis of acute cholecystitis in a duplicated gallbladder was established based on the clinical presentation and imaging findings. The patient underwent a laparoscopic cholecystectomy. Intraoperatively, a single large gallbladder was identified with a thick intraluminal septum and a solitary cystic duct (Figure 3), consistent with type I gallbladder duplication (septated gallbladder) according to the Harlaftis classification [8]. Intraoperatively, dissection of the biliary tree was challenging due to the presence of two closely approximated gallbladders sharing a common cystic duct. The distorted anatomy made it challenging to identify structures within Calot’s triangle, requiring cautious and deliberate dissection to avoid iatrogenic injury. The procedure was completed without complication.

**Figure 4:**
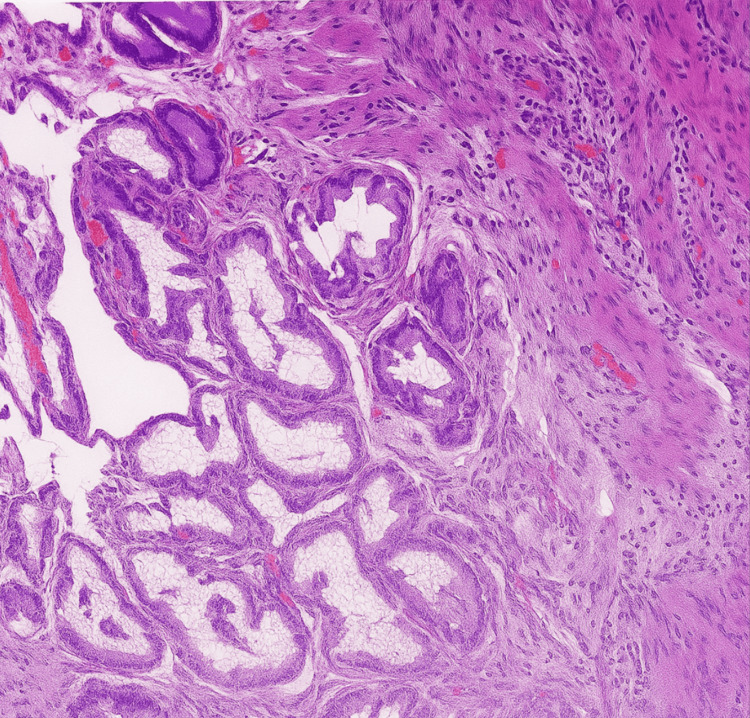
Hematoxylin and eosin-stained histologic section of gallbladder wall (30× magnification) H&E: hematoxylin and eosin Representative low-power H&E-stained section of the gallbladder wall showing features consistent with chronic cholecystitis. The mucosa is attenuated with focal lymphocytic infiltrates and areas of fibrosis within the muscularis layer. No evidence of epithelial dysplasia or malignancy is observed. These findings support the diagnosis of chronic inflammation in the setting of cholelithiasis

Tissue samples from both compartments of the gallbladder were submitted for pathological analysis. Gross and microscopic examination revealed an open, tan-colored gallbladder measuring 6.6 cm in length and 4.8 cm in circumference. The serosal surface was smooth but irregular. Sectioning revealed minimal yellow bile and a single yellow calculus measuring 2.0 × 1.2 × 1.2 cm. The stone had a yellow-brown, crystalline interior consistent with cholesterol composition. The gallbladder wall thickness measured up to 0.4 cm. The mucosal surface was slightly roughened but without significant abnormalities. Histologic sections (Figure 4) were negative for dysplasia or malignancy, with findings consistent with chronic cholecystitis and cholelithiasis.

The patient had an uneventful postoperative recovery, resumed a regular diet, and was discharged home in stable condition. Final pathology confirmed chronic inflammation with acute exacerbation of cholelithiasis in one of the duplicated gallbladders. At follow-up several weeks later, the patient reported complete resolution of symptoms and no postoperative complications.

## Discussion

**Figure 5:**
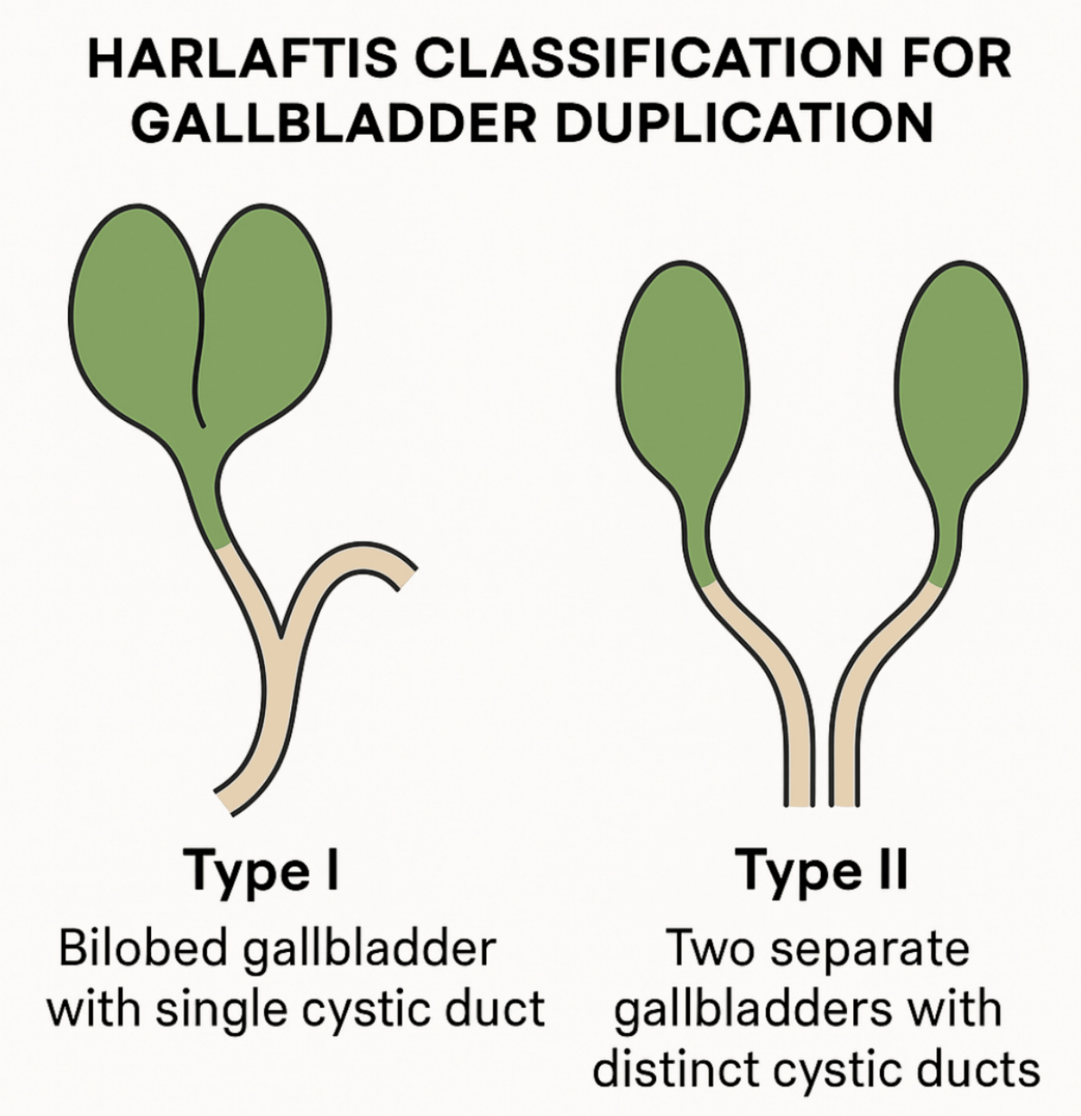
Harlaftis classification of gallbladder duplication Schematic representation of the Harlaftis classification for gallbladder duplication. Type I depicts a bilobed gallbladder with a single cystic duct. Type II shows two distinct gallbladders, each with its own cystic duct. This classification helps guide diagnostic interpretation and surgical planning. Figure created by the authors using OpenAI's ChatGPT image generation tool

Gallbladder duplication is an uncommon congenital anomaly that may be discovered incidentally during imaging or surgery, often complicating standard biliary procedures due to its variable anatomy [9]. While typically asymptomatic, it can significantly complicate the diagnosis and surgical management of biliary disease when associated with cholelithiasis or cholecystitis [10]. According to the Harlaftis classification (Figure 5), duplicated gallbladders are categorized into two main types. Type I involves a bilobed gallbladder with a single cystic duct, resulting from the early splitting of the cystic primordium, which produces either a septated or bilobed gallbladder. Type II consists of two entirely separate gallbladders with independent cystic ducts, arising from separate biliary diverticula [11]. The anomaly identified in our patient corresponded to a type I configuration.

Preoperative identification of duplicated gallbladders remains challenging, as imaging findings can mimic those of choledochal cysts, pericholecystic fluid collections, or a folded gallbladder [12,13]. In this case, although ultrasound and CT raised suspicion for atypical anatomy, definitive diagnosis was confirmed intraoperatively. A bilobed gallbladder with a shared cystic duct was identified, emphasizing the importance of intraoperative vigilance. Careful dissection and thorough exploration of Calot’s triangle were critical to avoiding inadvertent injury to the common bile duct or vascular structures and ensuring complete resection of the inflamed gallbladder [14].

Failure to identify such anatomical variations preoperatively or intraoperatively can result in incomplete cholecystectomy, persistent postoperative symptoms, bile duct injury, bile leakage, or the development of biliary strictures [12,14]. These complications may lead to significant postoperative morbidity, including peritonitis or the need for additional surgical or interventional procedures. In light of these risks, this case prompted a review of our preoperative imaging protocols to improve the detection of anatomic anomalies in patients presenting with recurrent or atypical biliary symptoms, thereby minimizing intraoperative or postoperative complications.

Histopathological examination of the resected specimen confirmed chronic cholecystitis, with no evidence of epithelial dysplasia or malignancy. The patient’s postoperative recovery was uneventful, with complete symptom resolution and no retained biliary structures identified on follow-up imaging. This favorable outcome highlights the critical role of intraoperative vigilance and accurate anatomical identification in the successful management of rare biliary anomalies.

This case highlights the clinical relevance of duplicated gallbladders and emphasizes the importance of maintaining a high index of suspicion in cases with atypical anatomy. Early recognition through advanced imaging, coupled with meticulous surgical technique, is essential for reducing operative risk and ensuring optimal outcomes [10,12]. Increased surgeon awareness of such anatomical anomalies and thoughtful use of preoperative imaging modalities can significantly enhance surgical planning and patient safety.

## Conclusions

Although rare, gallbladder duplication presents notable diagnostic and surgical challenges, particularly due to its anatomical variability. Clinical symptoms may mimic those of more common biliary pathologies, and unless specifically considered, this anomaly may go unrecognized until surgery. In cases of type I duplication, as presented here, the shared cystic duct anatomy can increase the risk of incomplete resection or biliary injury if not properly identified. Despite the limited sensitivity of current imaging modalities, this case underscores the value of thorough preoperative evaluation and intraoperative awareness. Timely recognition and careful dissection were crucial in achieving a favorable outcome. As such, this report emphasizes the importance of comprehensive planning and anatomical assessment to ensure safe and effective management of rare biliary anomalies.
